# The Many Faces of Covid-19: Organizing Pneumonia (OP) Pattern HRCT Features

**DOI:** 10.37825/2239-9747.1001

**Published:** 2020-10-01

**Authors:** G Rea, T Valente, R Lieto, G Bocchini, E Marchiori, A Pinto, A Maglio, A Vatrella

**Affiliations:** 1 Dipartimento di Radiologia, Ospedale Monaldi, A.O. dei Colli, Napoli, Italia; 2 Universidade Federal do Rio de Janeiro, Rio de Janeiro (RJ) Brazil; 3 Dipartimento di Radiologia, Ospedale CTO, A.O. dei Colli, Napoli, Italia; 4 Dipartimento di Medicina e Chirurgia, Sezione Malattie Apparato Respiratorio, Università di Salerno, Salerno, Italia

**Keywords:** Covid-19, GGO opacities, interstitial pneumonia, organizing pneumonia, HRCT

## Abstract

Covid-19 (coronavirus disease 2019) is an infectious disease caused by severe acute respiratory syndrome coronavirus 2 (SARS-CoV-2). On 30 January 2020 the World Health Organization (WHO) declared that the outbreak of Covid-19 realizes a public health emergency of international concern. Because of the primary involvement of the respiratory system, chest CT is strongly recommended in suspected Covid-19 cases, for both initial and follow-up. We present the case of a Covid-19 patient, a 57-year-old man, with a typical HRCT course of OP reaction.

Covid-19 (coronavirus disease 2019) is an infectious disease caused by severe acute respiratory syndrome coronavirus 2 (SARS-CoV-2). It is a member of the *Betacoronavirus* genus, one of the genera of the Coronaviridae family of single-stranded RNA viruses. The first cases were seen in Wuhan, China, in late December 2019 before spreading globally.[1,2] On 30 January 2020 the World Health Organization (WHO) declared that the outbreak of Covid-19 realizes a public health emergency of international concern. Because of the primary involvement of the respiratory system, chest CT is strongly recommended in suspected Covid-19 cases, for both initial and follow-up. [3] The primary Covid-19 pneumonia findings on CT in adults include ground glass opacities (GGO), and superimposed air space consolidations that are mostly bilateral, subpleural, peripheral, and usually basal in distribution. Traction bronchiectasis, crazy paving pattern and inter/intra-lobular septal thickening, halo sign, reversed halo sign, broncho-vascular thickening and vascular enlargement in GGO lesions are pulmonary associated findings that may be found in the various stages of the temporal evolution of the disease.[4] Atypical findings only seen in a small minority of patients (such as mediastinal lymphadenopathy, pleural effusions, tiny pulmonary nodules, tree-in-bud nodules, cavitations) should raise concern for a superimposed bacterial infection or others diagnoses. In some recent articles, it has been observed that in the intermediate stage (or peak stage, 9–13 days after the symptom onset) there is a progressive replacement of the same GGO areas by consolidations. The hypothesis of organizing pneumonia (OP) pattern as a response to viral damage is infrequently discussed among the kaleidoscopic patterns observed in Covid-19. Here, we present a Covid-19 patient with a typical HRCT course of OP reaction. A 57-year-old man presented to our Covid - Department with a 7-day history of dry cough. His medical history included hypertension, and mitral insufficiency treated with valve replacement ten years earlier. At hospital admission, blood oxygen saturation 92% in breathing room air and slight anosmia were present, without fever or arthro-myalgia. Physical examination showed a temperature of 36.7 °C, blood pressure of 155/105 mm Hg, heart rate of 78 beats/min, and rare mild bilateral posterior velcro-sounds. Laboratory tests showed a white blood cell count of 3.19 ×109/L with 75.3% neutrophils, 13.2% lymphocytes, and 1% eosinophils. Pro-calcitonin levels and C-Reactive protein were 45.7 mg/L and 0.85 ng/mL respectively. Cardiac enzyme levels, kidney and liver function were within the normal range. The SARS-CoV-2 nucleic acid tests were positive in throat swab samples two times and therefore for epidemiological characteristics and for these laboratory findings, Covid-19 pneumonia diagnosis was done. This patient underwent a baseline (T0) and (in a month) three (T1-T2-T3) sequential high resolution computed tomography (HRCT) of the chest. At baseline HRCT, bilateral lower lobes and posterior segment of upper left lobe peripheral extensive hazy GGO areas were visible, due to partial filling of airspace (Fig. 1A). At T1 follow-up HRCT time (11th day) consolidations replaced partially GGO areas, with posterior retraction of right fissure related to mild loss of lung volume (Fig. 1B). At T2 HRCT (18th day), the lung subpleural consolidations showed signs of resorption and reduction in extension; bubbly lucencies (representing unaffected aerated alveoli) and air bronchograms may be seen within the areas of consolidation (Fig. 1C). At T3 HRCT (30th day) slight bilateral parenchymal bands projecting from the visceral pleura towards the lung parenchyma in mild GGO areas were seen (Fig. 1D). These bands are usually up to 5 cm in length and 1–3 mm in thickness, and sometimes are suggestive of fine reticular fibrosing pattern such as in OP scars. OP is considered as an acute/sub-acute idiopathic interstitial pneumonia (IIP). When an underlying cause is unknown it is classified as cryptogenic organizing pneumonia (COP), whereas if a cause is known it is then termed a (secondary) OP.[5] For this reason, the diagnosis of COP should be made only after the exclusion of any possible etiology. Viral infections, drug use, connective tissue disease, toxic gases, aspiration of acid or oily substances, radiation therapy, chemotherapy, neoplasms, inflammatory bowel disease, and other causes can determine OP pattern.[6–9] OP equally affects both genders, typically in their fifth or sixth decade of life. This lung reaction has often a subacute respiratory course with a relatively short duration (median, less than 3 or 4 months) and the symptomatology of the patient can vary from modest dry cough with a fever of mild intensity and duration and asthenia. There is a group of patients who do not respond to corticosteroid therapy and develop a form of broncho-centric fibrosis with bronchial wall thickening and traction bronchiectasis different from the forms of pulmonary fibrosis in other IIPs. Recently in Covid-19 pneumonia, histopathological examination of lung biopsy tissues revealed prominent fibrous mucus exudate, organizing-phase DAD, and intra-alveolar loose fibrous plugs of OP.[10] Further autopsy study will serve to further clarify the damage in the lung and in the other target organs of the new coronavirus.

## Figures and Tables

**Fig. 1:**
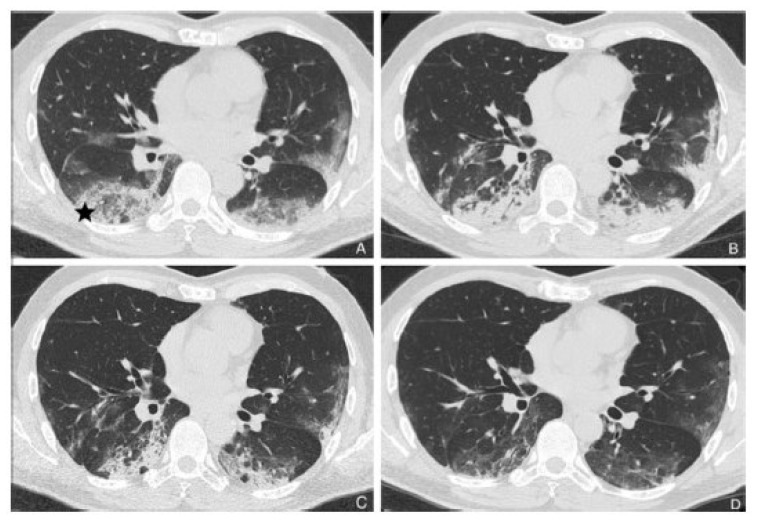
**A (T0: time 0):** axial HRTC scan of the chest at the level of the apical segments of the lower lobes showing the reversed halo sign (RHS) to right (star), as well as bilateral peripheral hazy GGO areas. **B (T1: 11^th^ day):** axial HRTC scan of the chest at the same anatomical level showing consolidations replace partially GGO areas. **C (T2: 18^th^ day):** axial HRTC scan of the chest at the same anatomical level showing partial reduction of extensive consolidations and two small bubble lucencies and air bronchogram in the areas of consolidations. **D (T3: 30^th^ day):** axial HRTC scan of the chest at the same anatomical level showing resorption of consolidations and some slight band-like elements within hazy GGO areas.
